# Formation of tungsten oxide nanostructures by laser pyrolysis: stars, fibres and spheres

**DOI:** 10.1186/1556-276X-6-166

**Published:** 2011-02-23

**Authors:** Malcolm Govender, Lerato Shikwambana, Bonex Wakufwa Mwakikunga, Elias Sideras-Haddad, Rudolph Marthinus Erasmus, Andrew Forbes

**Affiliations:** 1 CSIR National Laser Centre, P. O. Box 395, Pretoria 0001, South Africa; 2 School of Physics, University of the Witwatersrand, Private Bag 3, P. O. Wits 2050, Johannesburg, South Africa; 3 iThemba Labs, Private Bag 11, Wits 2050, Jan Smuts and Empire Road, Johannesburg, South Africa; 4 School of Physics, University of KwaZulu-Natal, Private Bag X54001, Durban 4000, South Africa

## Abstract

In this letter, the production of multi-phase WO_3 _and WO_3-__*x *_(where *x *could vary between 0.1 and 0.3) nanostructures synthesized by CO_2_-laser pyrolysis technique at varying laser wavelengths (9.22-10.82 mm) and power densities (17-110 W/cm^2^) is reported. The average spherical particle sizes for the wavelength variation samples ranged between 113 and 560 nm, and the average spherical particle sizes for power density variation samples ranged between 108 and 205 nm. Synthesis of W_18_O_49 _(= WO_2.72_) stars by this method is reported for the first time at a power density and wavelength of 2.2 kW/cm^2 ^and 10.6 μm, respectively. It was found that more concentrated starting precursors result in the growth of hierarchical structures such as stars, whereas dilute starting precursors result in the growth of simpler structures such as wires.

## Introduction

Tungsten trioxide is known as a 'smart material', because it exhibits excellent electrochromic, photochromic and gasochromic properties. Nano-sized tungsten trioxide has been applied in many nano-photonic devices for applications such as photo-electro-chromic windows [1], sensor devices [2,3] and optical modulation devices [4]. Many techniques for synthesizing nano-sized tungsten trioxide have been reported [5-8] and this article concerns with laser pyrolysis.

Laser pyrolysis is more advantageous than most methods because the experimental orientation does not allow the reactants to make contact with any side-walls, so that the products are of high quality and purity [9]. Laser pyrolysis is based on photon-induced chemical reactions, which is believed to rely on a resonant interaction between a laser beam's emission line and a precursor's absorption band, such that a photochemical reaction is activated [10]. The photochemical reaction enables an otherwise inaccessible reaction pathway towards a specific product, either by dissociation, ionization or isomerisation of the precursor compound. It was shown [8,11] that low laser power densities can also achieve the same desired products as the high power densities, presumably because of the way photon-energy is distributed into the energy levels of the precursor.

In this letter, the formation of W_18_O_49 _(= WO_2.72_) and the effect of the laser power, the wavelength on the morphology and structural properties of tungsten oxide nano-structured and thin films are reported.

## Experimental

**Figure 1:**
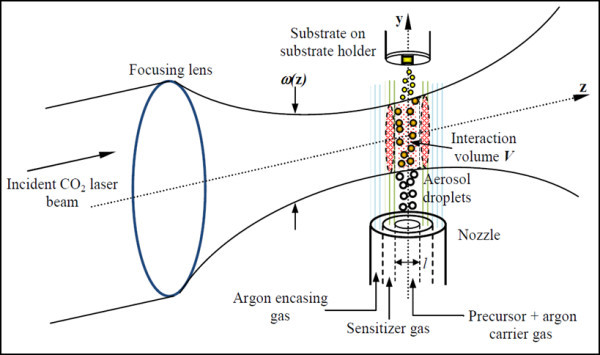
**A schematic of laser pyrolysis within the reaction chamber during laser-precursor interaction**.

The laser pyrolysis experimental setup was discussed in detail in [10], and a schematic description of the experiment during laser-precursor interaction is depicted in Figure 1. The laser pyrolysis method is carried out within a custom-made stainless steel chamber at atmospheric pressure. A wavelength tunable Continuous Wave CO_2 _laser was used in the experiments (Edinburgh Instruments, model PL6, 2 Bain Square, Kirkton Campus, Livingston, UK) and the beam was focused into the reaction chamber with a 1-m radius of curvature concave mirror which is effectively a lens with a focal length of 500 mm. For low power densities, an unfocused beam was used by replacing the concave mirror with a flat mirror. An IR-detector (Ophir-Spiricon, model PY-III-C-A, Ophir Distribution Center, Science-Based Industrial Park, Har Hotzvim, Jerusalem, Israel) was used to trace out the laser beam profile at various propagation distances from the flat or concave mirror to determine the beam properties.

The laser power was varied using a polarization-based attenuator, and the wavelength variation was achieved with an intra-cavity mounted grating in the laser. The different wavelengths were identified using a spectrum analyzer (Macken Instruments Inc., model 16A, Coffey Lane, Santa Rosa, California, USA) and the power output was measured with a power meter (Coherent Inc., 5100 Patrick Henry Drive, Santa Clara, CA 95054, USA).

The synthesis of WO_3 _and WO_3-__*x *_commenced by mixing 0.1 g of greyish-blue anhydrous tungsten hexachloride (WCl_6_, >99.9%, Sigma Aldrich, 3050 Spruce Street, St. Louis, MO 63103, USA) powder in 100 mL of absolute ethanol (C_2_H_5_OH, >99.9%, Sigma Aldrich) to give a tungsten ethoxide W(OC_2_H_5_)_6 _starting precursor [12]. Optical absorption properties of the precursor were determined using a Perkin Elmer Spotlight 400 FTIR Imaging System in the wavelength range 500-4000 cm^-1^.

The liquid precursor was decanted into an aerosol generator (Micro Mist, model EN, Research Triangle Park, NC 27709, USA) which was attached to the laser pyrolysis system via a multiflow nozzle that allows argon gas to carry the stream of very fine precursor droplets (5 μm droplet diameter according to the manufacturer) into the laser beam. Acetylene (C_2_H_2_) sensitizer gas and argon encasing gas flowed adjacent to the precursor, guiding it towards a substrate. The gas flow rates are chosen such that the ablated precursor collects on the substrate after interacting with the laser.

The sample was annealed for 17 h at 500°C under argon atmosphere [10]. Morphology studies were carried out using a Jeol JSM-5600 Scanning Electron Microscopy (SEM) microscope (using the secondary electron mode). Raman spectroscopy was carried out using a Jobin-Yvon T64000 Raman Spectrograph with a wavelength of 514.5 nm from an argon ion laser set at a laser power of 0.384 mW at the sample to minimize local heating of the sample during the Raman analysis. X-ray diffraction (XRD) was carried out using a Philips Xpert powder diffractometer equipped with a CuK_α _wavelength of 154.184 pm. The reproducibility of the experimental procedure was not verified.

## Results

**Figure 2:**
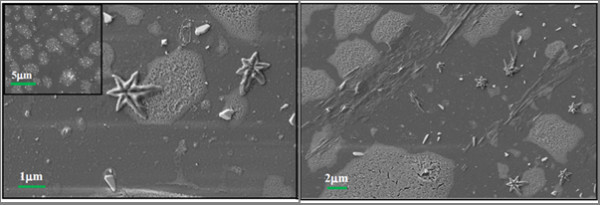
**Scanning electron micrographs of the post-annealed sample showing the growth of six-sided stars from the agglomerations of the pre-annealed sample depicted in the inset**.

When the CO_2 _laser beam was focused with a 1-m radius of curvature mirror, it produced a minimum beam radius or a beam waist of 1.2 mm, and at a laser power of 50 W on the 10.6-μm emission line, a power density of 2.2 kW/cm^2 ^was achieved. These parameters were consistent with those obtained when synthesizing WO_3 _nanowires using a very dilute precursor of 27 μM [10]. Laser pyrolysis of the more concentrated 2.5 mM precursor showed many uniform agglomerations composed of nanospheres (40 nm) before annealing, as depicted in the SEM micrograph in the inset of Figure 2. The sample was annealed, and from the agglomerates, stars grew with six points as seen in the SEM micrographs of Figure 2.

**Figure 3:**
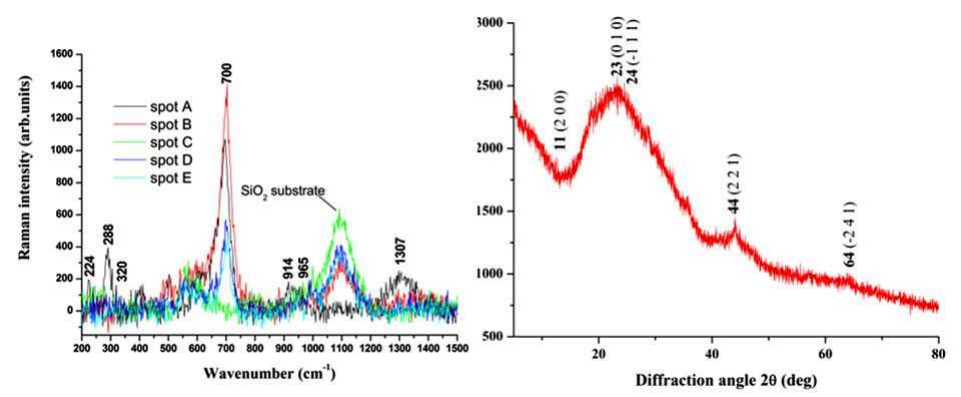
**Left: Raman spectrum and Right: XRD spectrum of the sample containing the stars**.

The Raman and XRD spectra of the samples containing the stars are shown in Figure 3. The stars were not visible under the Raman microscope and so various spots were analyzed on the sample. The Raman study shows that the sample is amorphous after annealing, and the lack of a dominant peak at 800 cm^-1 ^suggests the absence of monoclinic phase tungsten trioxide and possibly oxygen deficiency [13,14]. The Raman peaks found near 224 cm^-1^, 288 cm^-1 ^and 320 cm^-1 ^are indicative of a W-O-W stretching mode of a tungsten oxide. The Raman peak at 700 cm^-1 ^is designated to the bridging O-W-O vibrations in tungsten trioxide, and the asymmetry in this phonon peak shows that there are a number of phonons confined in the tungsten oxide layer of particles. This indicates that the product is composed of particles less than 20 nm in size [15,16]. The peak near 960 cm^-1 ^is assigned to the W^6+ ^= O symmetric stretching mode.

The XRD studies revealed peaks at 23° and 24° diffraction angles which suggests a tungsten oxide compound, but the lack of a triplet peak confirms the absence of monoclinic tungsten trioxide [17]. The broad hump at 22° resulted from SiO_2 _of the substrate, and this substantially decreased the signal-to-noise ratio making it difficult to identify the peaks. XRD peaks at 11, 40 and 64° diffraction angles are also evident in tungsten oxides [17], but the 44° diffraction angle suggests that the tungsten oxide has a deficiency of oxygen [18]. Based on the information from Raman spectroscopy and XRD, the most probable stoichiometry of this sample is monoclinic phase W_18_O_49 _(= WO_2.72_). According to the Powder Diffraction File (PDF 00-005-0392) that best matches the XRD spectrum in Figure 3, the lattice constants *a*, *b *and *c *are 18.28, 3.78 and 13.98 Å, respectively and the lattice angles are α = γ = 90° and β = 115.20°. The Miller indices are shown on the XRD spectrum in Figure 3.

Previously solid-vapour-solid (SVS) [8] and solution-liquid-solid (SLS) [19] mechanisms were proposed to explain the growth of nanowires of tungsten trioxide and platinum, respectively. Since the tungsten trioxide nanowires were grown with a low precursor concentration using a similar laser beam and laser parameters, the precursor concentration is seemingly the main contributor to hierarchical structure. This was confirmed by the 100 times more concentrated precursor that was used for the growth of the stars. The six-sided stars that were grown in Figure 2 looked very similar to lead (II) sulphide (PbS) stars that were grown by a concentration difference and gradient (CDG) technique [20]. This CDG technique used a high local concentration of one reactant mixed with a low concentration of another reactant under ambient conditions, where the high concentration favoured the thermodynamic conditions for crystal growth and the low concentration resulted in a diffusion-controlled kinetic environment for growth of hierarchical structures.

It is possible that due to a Gaussian laser beam profile, which has a high intensity at the beam's centre and low intensity at the edges, the region of intensity in the beam experienced by the precursor could vary the concentration of the decomposed material. It is speculated that this variation in concentration could have led to the growth of the hierarchical structures according to the CDG technique. The growth of stars has also been reported before for gold and molybdenum oxide [21,22], but not as yet for tungsten oxide. The literature proposes that star-shaped structures can be grown from agglomerates of more simple nanoforms under an inert atmosphere, which conditions were similar for this experiment [21,22]. One growth mechanism of nanostructures could be due to Gibbs-Thompson effect [9,23,24], which proposes that the size of the critical radius is dependent on the precursor concentration and explains the increase (Ostwald ripening) or decrease (Tiller's formula) in size of nanostructures.

The higher concentration probably provided a critical radius which resulted in simple nanoforms and the growth of stars as opposed to a lower concentration which resulted in microspheres and the growth of wires. It is speculated that the critical radius influences the thermodynamic and kinetic conditions as predicted by the CDG technique. Thus, the laser beam properties together with the relative precursor concentration contribute to the growth of stars. Some stars may form with four-sides and others with six-sides depending on the crystalline plane arrangement and the elements composing the structures [20]. It is not yet understood if the observed deficiency of oxygen plays a role in the formation of the six-sided stars or if the higher tungsten content, with a predominant valency of +6, has some correlation with the number of sides formed.

It is thought that acetylene gas acts as a photosensitizer [10] in laser pyrolysis, yet no evidence of absorption in the laser wavelength range 9.19-10.82 μm was found. This was verified by passing the acetylene gas through the laser beam at atmospheric pressure, and monitoring the power change during this interaction. The laser power did not appear to show any change, which implied that no radiation was absorbed by this gas. This does not, however, discount the possibility of some short-lived metastable state in acetylene induced by the laser which was undetectable by the power meter. The argon-precursor mixture, however, showed a change in power which indicated that the radiation was being absorbed, and the maximum absorbance was found at a wavelength of 9.54 μm. The absorbance of the precursor was given by the ratio of the laser power before laser-precursor interaction to the power observed during laser-precursor interaction.

**Figure 4:**
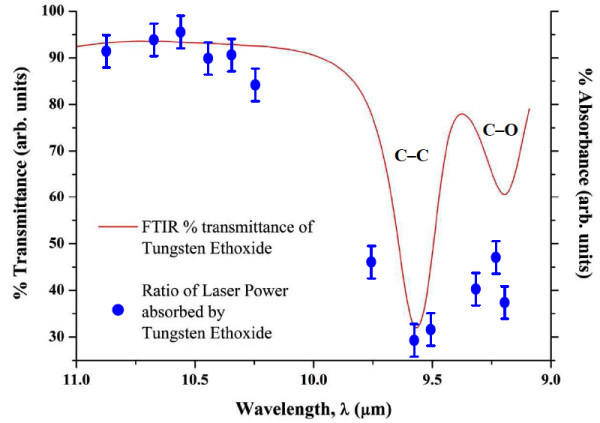
**The comparison of the FTIR transmittance spectrum of the tungsten ethoxide precursor and the CO_2 _laser radiation absorbance data of tungsten ethoxide as a function of wavelength**.

Figure 4 shows the absorbance by the precursor as a function of wavelength with the corresponding part of the FTIR transmission spectrum of tungsten ethoxide. This determination gives us an idea if the laser pyrolysis mechanism is a resonant process or if the precursor is decomposed by collisions with excited photosensitizer molecules. However, the results indicate that the laser energy gets transferred to the precursor and should cause decomposition by a resonant process, thus leading to the formation of the predicted products. Therefore, acetylene probably provides a reducing atmosphere in the laser-precursor interaction that influences the reaction pathway towards the formation of the products.

**Figure 5:**
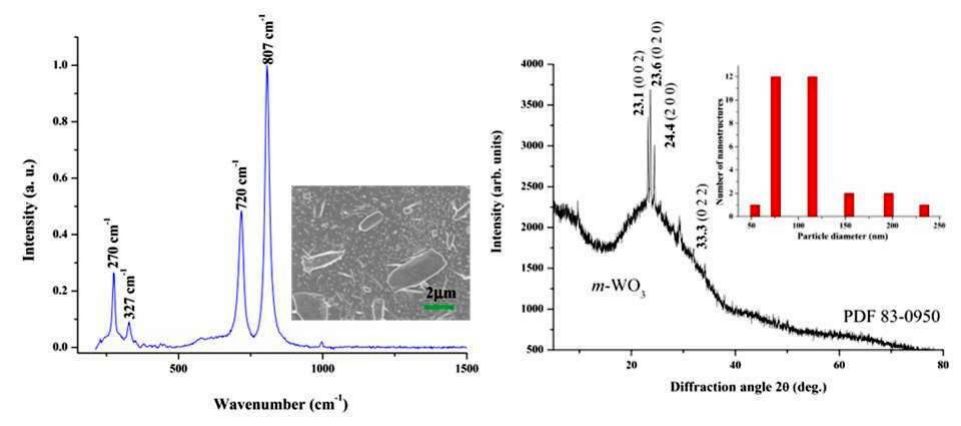
**Left: the Raman spectrum of the sample prepared at the 10.48-μm wavelength and 51.2 W/cm^2 ^power density with a SEM micrograph in the inset showing the morphology**. Right: the corresponding XRD spectrum with the histogram of the diameters of a selection of the nanostructures of the corresponding SEM micrograph in the inset. The Raman and XRD suggest a monoclinic phase WO_3_.

To determine how the laser wavelength plays a role in laser pyrolysis, it was varied between 9.19 and 10.84 μm at a constant power of 30 W and power density of 51.2 W/cm^2^. The lower power density was achieved by replacing the focusing mirror with a flat mirror to obtain a beam radius of 6.11 mm. The low power density was chosen such that all the *R*- and *P*-branches of the CO_2 _laser supplied a constant power output for the varying wavelengths. It was also assumed that at such low power density, minimum heating effects are involved in the laser-precursor interaction. It was found that only the 10.48-μm wavelength formed monoclinic phase WO_3 _according to the Raman and XRD spectra shown in Figure 5 with the corresponding SEM micrograph.

The nanosphere diameters of this sample, which were easiest to measure on SEM micrograph, were distributed in the range 50-250 nm as depicted in inset of Figure 5, and micron-sized fibres were also present in this sample. The theory speculates that if the laser wavelength is resonant with the C-O absorption band of the precursor (W-O-C_2_H_5_), then the C-O bond would break and lead to the formation of tungsten oxide. However, FTIR showed that the C-O absorption band is found between 9.00-9.38 μm (see Figure 4), and despite argon carrier gas presumably broadening the precursor absorption bands to some extent [25], the result could correspond to a non-resonant energy transfer. A 10.48-μm wavelength photon carries 0.1 eV of energy, and so 29 photons are required to dissociate a C-O bond [26] which corresponds to a multi-photon process. It is known, however, that tungsten ethoxide precursor can form WO_3 _upon heat treatment [12], which implies that the 10.48-μm wavelength could have had similar effects as annealing had. We believe that the shorter wavelengths, which had higher energy photons, dissociated various bonds which led to the formation of triclinic phase or a mixture of monoclinic and triclinic phase WO_3-__*x *_where *x *can vary between 0.1 and 0.3, depending on the laser parameters. It was also observed that the morphology of the samples became more randomized and of a disordered arrangement as the wavelength increased, and this is believed to be an effect of a corresponding decrease in energy.

**Figure 6:**
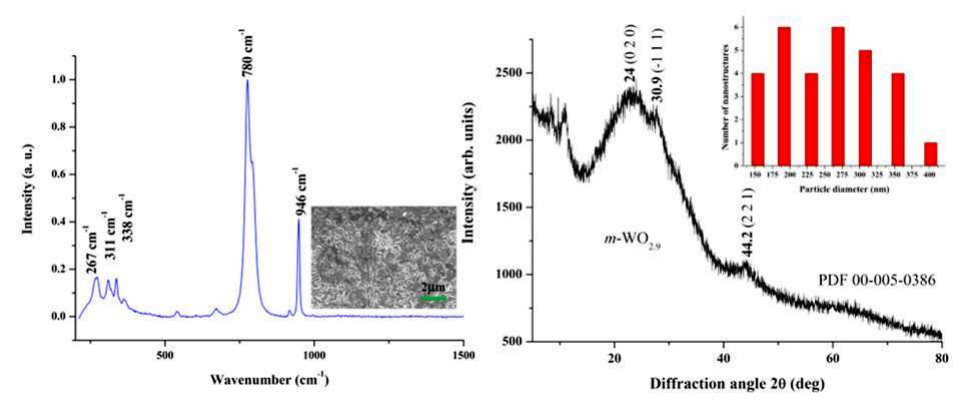
**Left: the Raman spectrum of the sample prepared at the 10.6-μm wavelength and 85 W/cm^2 ^power density with a SEM micrograph in the inset showing the morphology**. Right: the corresponding XRD spectrum with the histogram of the diameters of a selection of the nanostructures with the corresponding SEM micrograph in the inset. The Raman and XRD suggest a monoclinic phase WO_3-__*x*_(x~0.1).

Unlike the increasing wavelength, the increase in power density led to more ordered and shaped nanostructures, presumably because of the increase in energy rate. The 10.6 μm wavelength appeared to favour the formation of monoclinic phase tungsten oxide Furthermore, it was observed that at high enough power densities, it was more likely for helping nanostructure growth. At such low power densities (17-110 W/cm^2^) on the 10.6 μm wavelength, the particle sizes did not show a decrease with increasing power density as predicted [27] for the higher power density range (1-100 kW/cm^2^). The nanosphere diameters of this sample were found to be in the range 150-400 nm as depicted in inset of Figure 6. It was observed that the overall particle sizes were smaller for the power variation experiment, while the wavelength variation experiment showed larger particle sizes. The increase in power density did not always favour the formation of WO_3_, and since the photon energy was constant, only the number of photons per unit time varied.

Figure 6 shows the Raman and XRD spectra with the corresponding SEM micrograph of a sample prepared at a power density of 85 W/cm^2 ^at the 10.6-μm wavelength, which appeared to form a monoclinic phase WO_3 _according to the characteristic peaks.

Table 1 summarizes all the results obtained for the varying laser parameters. The average particle sizes observed for the wavelength variation was in the range 113-560 nm, while the average particle sizes for the power density variation were in the range 108-205 nm. The compositions of some samples were uncertain, and so it is written as WO_3-__*x *_where *x *most likely attains values between 0.1 and 0.3. There were no obvious trends as to how the laser parameters affected the product size or composition, and thus, it is believed that some possible competing reactions taking place during the laser-precursor interaction or during annealing.

**Table 1:** A summary of the results obtained for the laser power and wavelength variation

Wavelength Variation (P_density _= 51.2 W/cm^2^)			Power Density Variation (λ = 10.6 μm)		
***Wavelength, λ (mm)***	***Average sphere particle size (nm)***	***Composition***	***Power Density, P_peak _(W/cm^2^)***	***Average sphere particle size (nm)***	***Composition***

9.22	343	*m/t*-WO_3-__*x*_	17	157	*m*-WO_3-__*x*_
9.32	125	*m/t *-WO_3-__*x*_	26	122	*m*-WO_3-__*x*_
9.48	113	*m/t *-WO_3-__*x*_	34	140	*m*-WO_3_
9.70	403	*m/t*-WO_3-__*x*_	43	193	*m*-WO_3_
10.16	360	*t*-WO_3_	51	108	*m*-WO_3-__*x*_
10.36	560	*t*-WO_3_	60	122	*m*-WO_3-__*x*_
10.48	347	*m*-WO_3_	68	136	*m*-WO_3_
10.82	453	*t*-WO_3_	77	180	*m*-WO_3_
			85	205	*m*-WO_3-__*x*_
			94	114	*m*-WO_3-__*x*_
			100	106	*m*-WO_3_
			110	128	*m*-WO_3_
			2200	100	*m*-WO_2.72_

*M*, monoclinic phase; *t*, triclinic phase.

## Conclusion

Six-sided monoclinic phase WO_2.72 _stars were synthesized by laser pyrolysis technique using a more concentrated starting precursor and near-Gaussian laser beam profile. The higher concentrated precursors are required to obtain hierarchical structures as predicted by the literature. Laser wavelengths above 10 μm seem to favour the formation of stoichiometric WO_3_, but only at certain power densities, presumably to overcome possible competing reactions. Owing to the nature of photochemical reactions and the many stoichiometries and multi-phases that tungsten oxides can form, some product compositions were written as WO_3-__*x *_where *x *most probably assumes values between 0.1 and 0.3. The higher power densities were found to be essential for the further growth of structures and for smaller particle sizes. The authors now have an idea of the possible shapes of nanostructures that can be synthesized with possible chemical compositions, and the determination of the electrical and optical properties of these structures to observe possible unique characteristics allows for the tailoring of sensor devices that operate at room temperature for example.

## Conflict of interest

The authors declare that they have no conflict of interests.

## Authors' contributions

MG carried out all the experiments in conjunction with LS. MG also initiated the first draft manuscript. BWM assisted with the production of thin films by laser pyrolysis, characterization, analysis, interpretation of experimental results and manuscript handling. AF performed the optical alignment of the laser pyrolysis and discussion of the manuscript. ESH contributed through discussion of the manuscript and RE provided the Raman spectral data from all samples.
